# Characterization System for Heat-Energy to Electric-Energy Conversion from Concrete by Means of a Thermoelectric Module

**DOI:** 10.3390/s22051881

**Published:** 2022-02-28

**Authors:** Luis C. Félix-Herrán, Alejandro García-Juárez, Luis Arturo García-Delgado, Pablo Said González-Aguayo, Jorge de-J. Lozoya-Santos, José R. Noriega

**Affiliations:** 1 Tecnologico de Monterrey, School of Engineering and Sciences, Blvd. Enrique Mazón López 965, Hermosillo 83000, Sonora, Mexico; lcfelix@tec.mx; 2 Departamento de Investigación en Física, Universidad de Sonora, Blvd. Luis Encinas y Rosales, Hermosillo 83000, Sonora, Mexico; alejandro.garcia@unison.mx (A.G.-J.); arturo.garcia@unison.mx (L.A.G.-D.); pablosaidga@gmail.com (P.S.G.-A.); 3 Tecnologico de Monterrey, School of Engineering and Sciences, Ave. Eugenio Garza Sada 2501, Monterrey 64849, Nuevo León, Mexico; jorge.lozoya@tec.mx

**Keywords:** energy management, smart sensors, characterization system

## Abstract

The present work describes the implementation of a prototype to characterize thermoelectric modules (TEM). The goal is to study the energy conversion by means of thermoelectric modules mounted on concrete structures. The proposed experimental system is used for the electrical characterization of a commercially available thermoelectric module TEC1-12710 to prove its operation while embedded in a concrete slab, typical of building constructions. In this case, the parameters that define thermal energy conversion into electrical energy are open-circuit voltage generation, loaded circuit voltage generation, and load current. A known external load is connected to the terminals of the TEM for the purpose of its electric characterization. An electrical heating element on the hot side and a thermoelectric cooler on the cold side produce a temperature difference on the concrete slab. This arrangement allows the emulation of a temperature gradient produced by sunlight over a concrete structure. The objective is to measure the resulting electrical energy produced by the combination of concrete slab and the thermoelectric module. By controlling the temperature difference between the sides of the thermoelectric module under test, it is possible to simulate the effect of the temperature gradient under different sunlight conditions. Two digital PI controllers regulate the temperature conditions, thus providing controlled conditions for the experiments.

## 1. Introduction

One of the most attractive renewable energy sources is solar energy because of its availability, inexhaustibility, and accessibility. The amount of solar radiation varies according to various factors, some of which are listed in [1,2,3]. This resource is ecologically essential and financially crucial because it triggers economic growth in the region, so the motivation and relevance of using solar radiation increases.

Solar energy is used in two ways, heat or light, to convert it into usable electrical energy. Perhaps the most popular one is electrical energy that employs solar cells; however, thermal energy is also an attractive option whose application could be exploited in regions with high solar radiation and extreme temperatures. In general, the thermal energy from the sun is channeled through heliostats that concentrate the sun’s rays on a heat retainer body to obtain very high temperatures and thereby convert the heat into electrical energy [4], which can be employed to power sensors in diverse sustainable applications [5,6,7,8]. Solar heat can be stored for hours after sunlight is over within a mass of refractory material. Two main disadvantages of thermal energy from the sun are evident. The first one is that it is only available during daylight. The second one, the infrastructure needed to concentrate solar radiation to heat up a solid or liquid, is expensive and requires extensive maintenance [9,10,11,12]. Most heat energy is usually transformed into electricity by electromechanical means, implying the use of complex infrastructure. Other applications of heat energy are building heating and hot water supplies.

**Figure 1:**
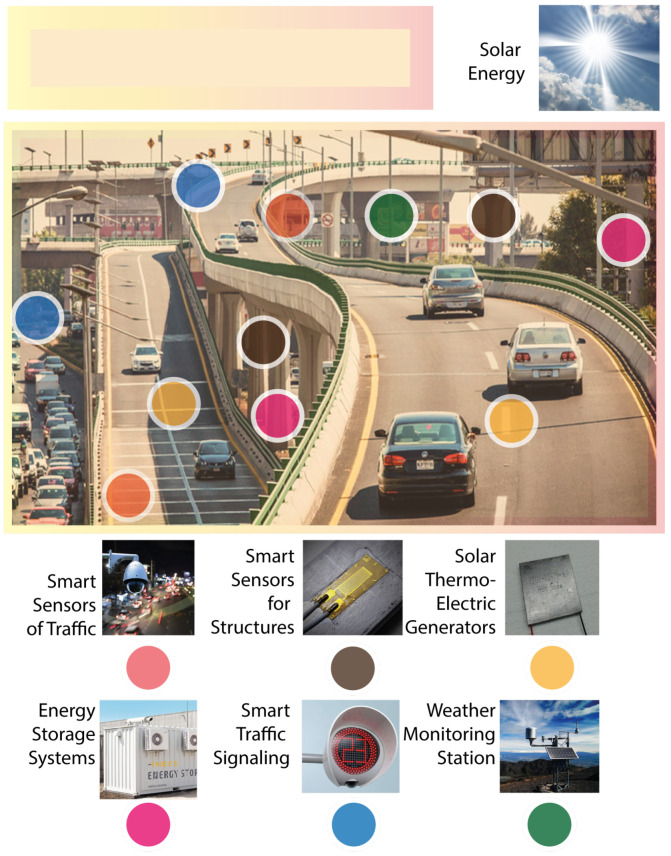
Smart system example for the management of traffic using solar thermoelectric generation in urban highways.

Most city buildings and infrastructure are constructed using concrete, which stores solar energy in the form of heat [13,14,15]. Moreover, the roofs of houses, buildings, and industrial buildings are examples of concrete structures that store heat during the day. Other significant structures outside cities are also built using concrete, such as highways, bridges, and dams, to mention a few. Such structures store large amounts of heat for hours after sunlight is over. However, the heat stored in these structures is not used in practical applications, and it is usually lost (Figure 1).

Thermoelectric modules are maintenance-free semiconductor devices, which can be used to transform the thermal energy stored within concrete structures and, in some applications, may be an approach to complement other renewable energy conversion systems such as heliostat mirror fields [16,17]. Thermoelectric modules are usually applied for cooling; by passing a current through it, one side of the device will cool down, while the other side will heat up. When one of the sides of a thermoelectric module is heated up while the other is kept to a lower temperature, the device will convert the heat flow partially into electric energy. The operation of these devices is based on the Seebeck effect, which states that an electromotive force is developed on a conductive material when a temperature difference is applied to both sides of the material in question [18]. A disadvantage of this energy-conversion method is its efficiency. The efficiency of thermoelectric modules as energy converters is low [19,20]. Some thermoelectric modules in the market are designed for energy conversion but usually operate efficiently above 100 ${}^{\circ }$C, with a cold side temperature of around 50 ${}^{\circ }$C [21]. Nevertheless, at warm latitudes (35${}^{\circ }$ S to 35${}^{\circ }$ N), some concrete structures can reach a temperature at the sunny side close to 80 ${}^{\circ }$C, during the daytime, and a temperature at the shadow side of about 30 ${}^{\circ }$C. This temperature difference is within the operating range of the thermoelectric modules [22].

This work aims to propose a simple mechanism for the characterization of assemblies of thermoelectric modules embedded into concrete samples. In order to determine all valuable figures of merit, electrical, thermal, energy generation, and performance, the assemblies must be subjected to temperature differences under controlled conditions. The characterization device proposed here must emulate the effect of sunlight and heat on one side of the slab and the temperature difference of the shadow side of the slab. Additionally, combinations of additive materials mixed with concrete and their effects on energy storage and conversion can be tested. The system described here consists of two electrical resistors used to heat one side of a cubic slab of concrete of 10 cm × 10 cm × 10 cm, a thermoelectric device attached to the opposite side of the heated side of the slab, and a cooling module attached to the opposite side of the thermoelectric module undergoing testing (TMUT). The resistors will heat the sides of the slab, emulating the effect of direct solar irradiation, and the thermoelectric cooler is used to emulate the temperature on the shadow side of a concrete structure, thus creating a temperature difference on both sides of the thermoelectric module being tested. The rest of the article is organized as follows: Section 2 is a description of the prototype components, design, and operation; Section 3 describes the mathematical modeling, design, and simulation of the prototype; Section 4 develops the closed-loop system description; Section 5 describes the experimental arrangement, tests, and results, and, finally, Section 6 presents conclusions and proposed further work.

## 2. The Prototype—Design and Implementation

### 2.1. Conceptualization

**Figure 2:**
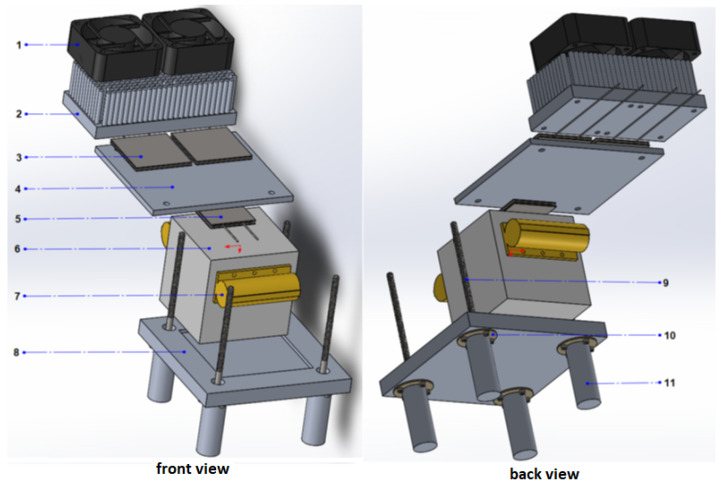
Exploded front and back view of the prototype.

To produce a temperature difference on the concrete slab, a couple of heater resistors are attached to the slab on opposing sides, as can be seen in Figure 2. These resistors, labeled number 7 in Figure 2, emulate the effect of solar irradiation over the exposed sides of the concrete slab, as it commonly occurs on a concrete structure. One of the sides lodges the thermoelectric module under test (number 5 in the figure), having the module’s hot side directly in contact with the concrete slab. The heat from the concrete slab must flow unimpeded to the hot side of the thermoelectric module. Thus, the thermoelectric module is mechanically attached to the concrete slab, and heat transfer is improved by applying thermoconductive grease used for heatsinks. Although the effectiveness of the thermoconductive grease was not tested, it appears to help in the heat transfer process. Hence, materials must be investigated to optimize heat transfer between concrete and the ceramic side of the thermoelectric modules. The cold side of the thermoelectric module is attached to an aluminum plate (number 4 in Figure 2), which is also attached to a couple of thermoelectric modules, heatsink, and fans, for cooling. Thus, the aluminum plate emulates a concrete structure’s cold side or shadow side. The rest of the prototype components are required for mechanical attachment and thermal insulation to reduce potential thermal disturbances. In particular, polyurethane insulation was placed between the concrete slab and the aluminum plate of the cold side, in order to avoid an undesired exchange of heat. This section covers the design aspects of the prototype, which include the schematics, the integration of the components, electronics, and the assembled prototype.

On one of its sides, the hot element is attached to the thermoelectric module, and another two sides are in contact with electrical heating resistors. These last elements act as actuators to heat the concrete cube. Furthermore, to reduce the heat loss from the heating resistors to the environment and to achieve higher temperatures, polyurethane foam was used on the remaining sides of the cube. Moreover, the cold element is an aluminum plate, which is in contact with the thermoelectric module on one of its faces. On the other side of the cold element, two cooling Peltier cells transfer the heat to the air through the heatsinks.

### 2.2. Computer-Aided Design (CAD) and Integration

The mechanical components of the prototype were designed using SolidWorks^TM^ CAD software version 2019. The complete design, front, and back views are seen in Figure 2. The main components of the prototype are listed below.

List of mechanical components illustrated in Figure 2, the following elements are depicted:

1. Cooler fans.
2. Heatsinks.
3. Peltier modules for cooling (TEC1-12715).
4. Cold element (aluminum plate).
5. Thermo-electric module under test (TMUT).
6. Concrete slab.
7. Thermal resistors (HS100 2R7 J).
8. Aluminum support.
9. Threaded rods to hold the upper section.
10. Insulating plate.
11. Aluminum supporting rods.

In Figure 2, the slab is made of a conventional concrete type II, also known as Portland Cement, which is commonly used in building constructions. The slab was designed to be a cube of 10 cm × 10 cm × 10 cm, thus having an approximate volume of 1 L or $1\times 10^{-3}\;m^{3}$. This small volume was selected to keep a low energy requirement during testing. To reduce a potential parasitic heat exchange, thermal insulation was applied around the concrete cube, which is not indicated in Figure 2. In the initial design, the heating resistors were placed on the opposing side of the concrete slab to simulate direct sunlight on the concrete slab, thus heating the slab bottom side. However, using this arrangement, a substantial delay affects heat transfer from the heated upside to the opposing side of the concrete slab, where the TMUT is located, unnecessarily lengthening the experiment’s time. Thus, the heating resistors were positioned closer to the top of the concrete slab to reduce the delay. However, a time delay of several minutes still exists, which affects the temperature control of the slab. Moreover, the rest of the prototype is held in place by threaded rods and nuts.

**Figure 3:**
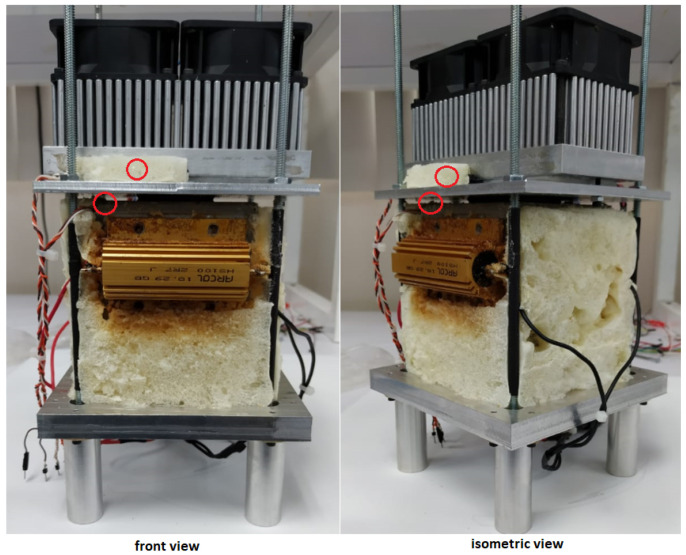
Assembled prototype, the polyurethane insulation was removed to expose some of the components. The red circles indicate the position of the temperature sensors.

Most of the components were machined from aluminum, and the heat insulators were machined from FR4 (fiberglass-reinforced epoxy-laminated) sheets. The manufactured prototype, from the design in Figure 2, is shown in Figure 3. At this point, the prototype is fixed in an upright position, but it can be repositioned for the emulation of different conditions of sunlight irradiation and wind currents.

For a better understanding of the manufactured prototype, descriptions, dimensions, and quantities of the components are shown in Table 1.

**Figure 4:**
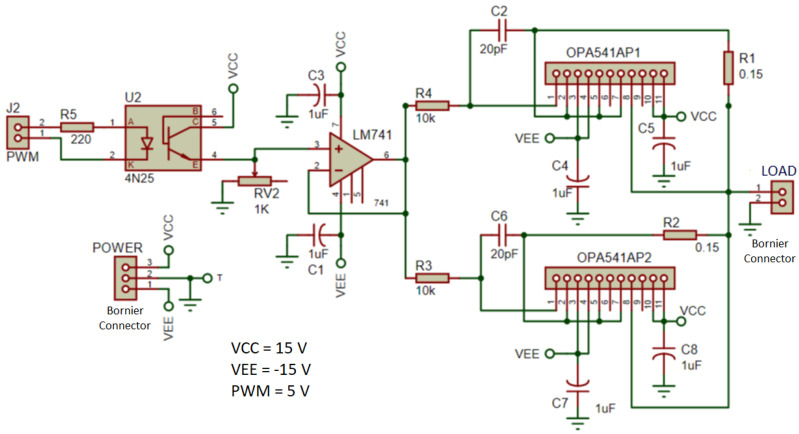
Schematic of the two power driver circuits. This driver is used to supply power to the heating resistors and the thermoelectric modules for cooling. VCC = +15V, VEE = −15V, and PWM = +5V.

The printed circuit boards (PCB) for the thermoelectric modules drivers were designed using PROTEUS software for schematic capture and PCB design. The PCBs for the amplifiers were manufactured in-house using a PROTOMAT H100 from LPKF. The schematic circuit of the drivers for the thermoelectric modules is illustrated in Figure 4. A list of the main components of the circuit are included in Table 2.

For safety reasons, the bornier terminal block connectors illustrated in Figure 4 were abandoned in favor of soldering the high-current-carrying cables directly to the PCB.

### 2.3. Power Supply and Power Circuit

The heating resistors require high currents to heat the concrete slab, supplied by a pulse width modulation (PWM) driver circuit. In addition, the cooling thermoelectric modules also require high currents to operate; thus, driver circuits were designed and implemented to work as power interfaces between the microcontroller and the power supplies for the heating resistors and the cooling thermoelectric modules. The diagram of the driver circuits is illustrated in Figure 4, where VCC is linked to the microcontroller in the following manner; while the microcontroller is fed with 5 V, the Peltier modules are supplied with 15 V.

In the schematic shown in Figure 4, the driver circuit is composed of two high-output current operational amplifiers (op-amp) OPA541 and one LM741 op-amp; Texas Instruments manufacture both integrated circuits (IC). In addition, various resistors and decoupling capacitors were also included in the design. Moreover, the circuit was completed by an optocoupler 4N25 manufactured by Vishay Semiconductors. The power op-amps were connected in a unit follower configuration to transfer the PWM voltage to the LOAD. A current limiting resistor was used for each power op-amp, namely R1 and R2.

The optocoupler 4N25 provides a safe separation of grounds between the PWM circuit generator and the power driver circuit, to protect the microcontroller. The unit follower, implemented by the LM741, drives the two OPA541 high-current op-amps, which supply high-current PWM pulses to the heating resistors. A similar driver circuit drives the cooling thermoelectric modules for the cold side. As for the heating resistors, a PWM signal is applied to drive the cooling thermoelectric modules. In order to supply the needed power, two switching power supplies were used. The current pulses can reach a peak close to 10 A, which is the maximum output current of the power driving circuit. Furthermore, a pulse width modulated signal, or PWM, is fed to the driver through the optocoupler to produce high-current PWM pulses to pass through the cooling thermoelectric modules.

It is important to mention that two Peltier modules TEC1-12715 were used for cooling of the cold plate. These modules have a maximum temperature difference of 70 ${}^{\circ }$C, a maximum voltage of 16 V, and a maximum current of 15 A, with a maximum cooling capacity of 150 W. The ARDUINO UNO^®^ board is fitted with six PWM signal generators, which do not require additional circuitry to work. Therefore, using a PWM was convenient. For the ARDUINO board, the PWM duty cycle goes from 0% to 100%, meaning that the PWM signal ranges from 0 V DC up to full 10 V DC. Thus, the condition of maximum driving power for the cooling Peltier modules is continuous DC and not PWM. Hence, at 100% duty cycle, the cooling modules are driven with the maximum electrical current of 10 A. Since both Peltier modules are connected in a parallel arrangement, this current is split in two. According to the datasheet, under this condition, each module possesses a maximum cooling capacity of around 25 W.

An ARDUINO UNO^®^ microcontroller was used as a digital interface for the acquisition of analog signals for temperature measurements, the generation of digital control signals, and for the generation of PWM signals for the power driver module. Measurements of temperature from the hot side of the concrete slab and the cold side of the TMUT are used as feedback for digital control of each side’s temperatures. Thus, a temperature difference can be created and kept constant by controlling the temperature on the hot and cold sides of the TMUT. The digital controller was implemented using LabVIEW in a general-purpose PC.

More design aspects for the power interface module are described as follows:

- Since the microcontroller can deliver up to 5V and the loads that are required demand voltages above this value, the potentiometer RV2 was used to adjust the maximum voltage that goes to the power section.
- Tantalum capacitors were chosen for source decoupling (C1, C3, C4, C5, C7, C8), and also the ceramic capacitors C2 and C6.
- Resistors R1 and R2 are current limiters. For the experiment, it was limited to 4.78A, because the maximum current that the OPA541APs can continuously support is 5A. The sum of the total current from the two amplifiers towards the load is 9.56A.

The circuit gain is controlled by the potentiometer RV2, which controls the voltage level applied to the load. The load can be either the heating resistors or the cooling thermoelectric modules. The potentiometer RV2 forms an adjustable resistor whose output voltage depends on the collector current of the phototransistor inside the 4N25 optocoupler. The collector current ($I_{CE}$) in the 4N25 is dependent on the internal LED current ($I_{F}$) and temperature. The ratio of the collector current vs. the LED current ($I_{CE}$/$I_{F}$) assumes three different values for the operational range of the 4N25, at a given temperature according to *Figure 6* (collector emitter current vs. LED current, $I_{F}$ vs. $I_{CE}$) in the datasheet [23]. Therefore, there are two intervals for voltage output from the optocoupler–potentiometer arrangement. By inspection of the plot for $I_{f}$ vs. $I_{CE}$ from the datasheet of the 4N25 (Vishay Semiconductors), it can be found that

(1) $$I_{c}=\alpha_{1,2}\times I_{LED}$$

where $\alpha_{1}=0.8$, for the case when the LED current in U2 is $I_{LED}<10$ mA, and $\alpha_{2}=0.7$, for the case when the LED current is 10 mA $<I_{LED}<30$ mA. Since the peak PWM voltage produced by the ARDUINO UNO^®^ microcontroller is 5 V, the peak $I_{LED}$ current is computed from

(2) $$I_{LEDpeak}=\frac{5\;V}{220\;\Omega }$$

The PWM voltage that is passed on to the LM741 can be computed from

(3) $$V_{PWMpeak}\approx \mathrm{RV}2\times \alpha_{1,2}\times I_{LED}$$

Therefore, by adjusting the value of RV2, we have control over the amplitude of the PWM voltage supplied to the load. The LM741 and OPA541 op-amps are arranged in unity follower configuration, not affecting the output voltage amplitude. By adjusting the duty cycle of the PWM signal, the average power supplied to either load is controlled. This is achieved by changing the PWM duty cycle register in the ARDUINO UNO^®^ microcontroller.

Two limiting resistors, one for each OPA541 op-amps, are connected to the op-amps outputs. The limiting resistor values are calculated to avoid overheating the devices and guarantee that enough current is supplied to the heating resistors or the cooling thermoelectric modules. The maximum current expected to be drawn by the load from the OPA541 has been defined as 5 A. Therefore, the maximum current supplied by each power driving circuit is 10 A. The value of the limiting resistors R1 and R2 is calculated from

(4) $$R1,2=\frac{0.813}{|I_{lim}|}-0.02$$

Computation of the limiting resistor according to this equation leads to R1,2=0.1426Ω. The closest commercial value available is 0.15Ω, which is the value of the limiting resistors R1 and R2 used in this work.

### 2.4. Complete Prototype

**Figure 5:**
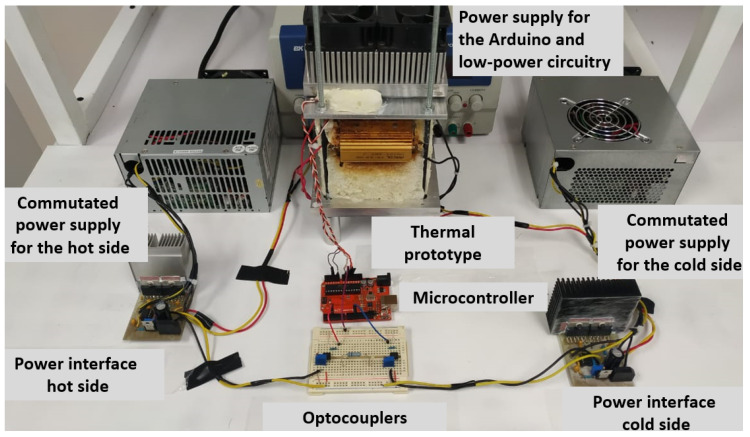
Complete implemented prototype.

The thermal module, power circuits, supply sources, optocouplers, and the microcontroller were interconnected to obtain the complete prototype, as shown in Figure 5. It is worthwhile to mention that the prototype was designed for the purpose of a later characterization of the heat energy conversion into electrical energy by a TMUT in contact with a concrete slab. At the moment, the present work focuses on temperature control and the demonstration of the basic functionality of the setup. The most important features of this design are the possibility to explore improved materials for heat transfer, and how heat energy can be converted directly into electricity (clean energy).

The prototype proposed in this work has been designed to operate close to the temperature range of 0–80 ${}^{\circ }$C. It is expected that solar irradiation will heat up the exposed concrete of a building within this temperature range. Thermocouples and thermistors have much wider temperature ranges, and are not necessarily linear within the temperature range required. Additionally, thermocouple voltage will vary with time, drifting out of tolerance. Sensor linearity is important in order to avoid unnecessary complexity in the design, reduce systematic errors, and to avoid linearizing algorithms. On the one hand, according to the Texas Instrument (TI) datasheet of the LM35, accuracy of the sensor is 0.5 ${}^{\circ }$C at 25 ${}^{\circ }$C. On the other hand, the T-type thermocouple, which is the most appropriate for the proposed temperature range, possesses 1.0 ${}^{\circ }$C standard limit of error at the required temperature range. Although cost is not an important issue, the solution proposed using the LM35 is inexpensive when compared to a thermocouple. Additionally, LM35 sensors are widely available. In spite of the small form of LM35 sensors, the time constant is larger than that of a thermocouple, resulting in a lagging response when compared to the response of a thermocouple. Nevertheless, the time delay introduced by the LM35 sensor is only of a few seconds and the effect on the measurement and the closed loop control can be neglected. Therefore, it was decided that the LM35 was the appropriate choice of sensor for the prototype. Both sensors were installed as closed as possible to the interface created by the faces of the TMUT with the cold and hot sides. Heat conductive compound was used for both LM35 sensors to improve heat transfer, although its effectivity in this case has not been tested. Both sensors were protected from other sources of heat using polyurethane foam. Inhomogeneities of temperature and heat flux have not yet been investigated. However, polyurethane foam was used to insulate the cold side, from the heat of the concrete slab, in order to minimize some possible heat exchange.

## 3. System Modeling

In order to characterize a TMUT, it must be subjected to a constant temperature difference, which must be adjustable over a specific temperature range. Therefore, an automatic control algorithm must be designed and implemented for this purpose. Modeling the heat exchange between the hot side and the cold side is the first step in designing and implementing the control algorithm needed to achieve a controlled temperature difference, as indicated above. This section describes the mathematical equations that model heating and cooling dynamics involved in the operation of the instrument. These are differential equations for the hot side temperature ($T_{hs}$) and the cold side temperature ($T_{sc}$). Laplace domain variables or transfer functions are used for a short notation, where appropriate.

**Figure 6:**
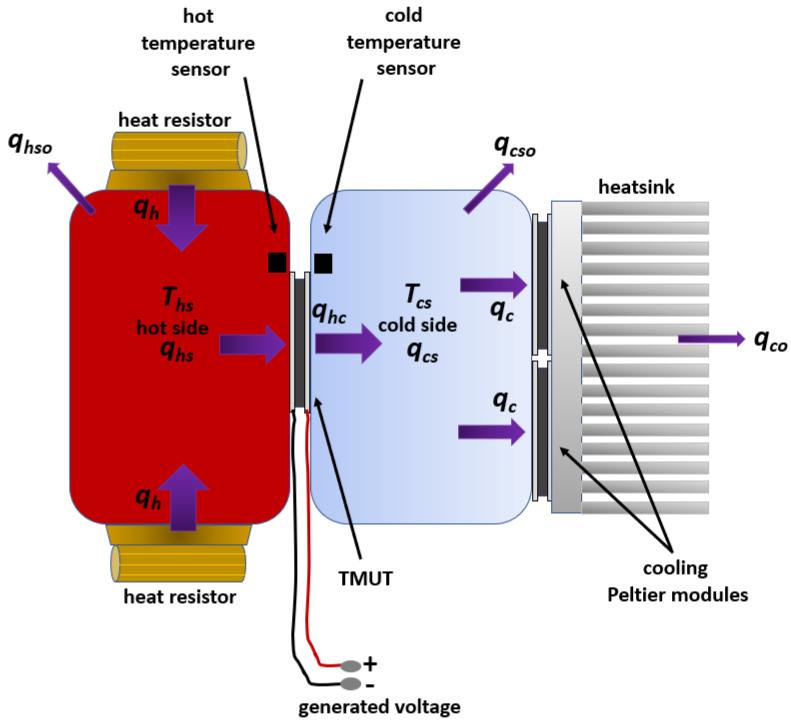
Schematic of the heat flow from the heat resistors, through the hot and cold sides, as well as the Peltier element, and out of the system by means of the heatsink.

As previously explained, the heat transfer in the prototype is performed by thermal conduction. The heat is generated by the heating resistors, represented by $q_{h}$, and transferred through the Peltier cell as $q_{hc}$ towards and across the cold side. The heat, which is transferred to the cold side of the assembly, moves out of the cold side as $q_{c}$, and it is dissipated as $q_{co}$ to the environment by the cooling fans. It is important to mention that some heat is stored and distributed in the concrete cubes, i.e., $q_{hs}$ on the hot side, and $q_{cs}$ on the cold side. Moreover, some stored heat in the hot and cold sides is lost ($q_{hso}$ and $q_{cso}$) when transferred to the environment. The schematic and individual heat flow contributions are shown in Figure 6.

The design of the temperature control algorithms for the hot and cold side requires balanced equations of heat conduction. The aim of the hot side controller is to reach a heat balance in the concrete slab among the heat provided by the heating resistances $q_{h}$, the heat flowing out through the TMUT $q_{hc}$, and the heat stored in the concrete slab $q_{hs}$. This heat balance leads to a constant temperature in the hot side. For the temperature controller of the cold plate, the aim is to reach a heat balance among the heat leaking through the TMUT into the cold plate, the heat flowing to the air from the heatsink, and the heat stored in the cold plate. The resulting heat balance manifests as the steady temperature of the cold side. The temperature controller for the cold side and the controller of the hot side work independently of each other. Therefore, the equations for heat conduction do not consider internal heat resistances of the TMUT. Hence, in order to produce a constant temperature difference on the TMUT faces, the controller algorithms only require data from the temperature at the interface of the TMUT with the concrete slab and with the cold plate. By inspection of Figure 6 and assuming no other sources of heat or heat leaks, the heat flow is modeled as follows:

(5) $$q=K\Delta T=\frac{kA}{x}\Delta T$$

In Equation (5) the heat flow *q* is measured in W; additionally, *K* is the thermal conduction coefficient in $W{/}^{\circ }C$, whereas *k* stands for thermal conductivity in $W/m^{2}{\;}^{\circ }C$. Moreover, *A* represents the area, $m^{2}$; *x* is the Peltier TMUT’s thickness in m, and ΔT symbolizes a temperature difference, measured in ${}^{\circ }C$ (all temperature differences and measurements are expressed in ${}^{\circ }C$). For more information about the heat flow dynamics, refer to [17,24]. Here, it is assumed that the TMUT is a homogeneous structure and that heat passing through it flows in perfect fashion (Fourier heat conduction). This assumption is relevant for the design of the controllers. Since the TMUT will operate to transform heat energy into electric energy, in a sub-volt range, no large electrical currents would flow through it and it has been assumed that self-heating could be neglected (Joule effect). The TMUT used in the experiments is a completely sealed Peltier device TEC1-12710. Although different thermal resistances are present inside of the TMUT, it was assumed that the device was a homogeneous structure in order to simplify the design and analysis of the controller equations.

Based on the heat flow shown in Figure 6, the flow of heat is modeled approximately, assuming only that the flow effectively moves across the concrete slab, the thermoelectric module, and the cold face, as if it were a homogeneous body. This first modeling approximation allows the testing of the control stages and it will be reformulated in further experiments. Based on this assumption, the hot side can be modeled as follows:

(6) $$q_{hs}=q_{h}-q_{hc}$$

In Equation (6), $q_{hs}$ represents the heat flow stored in the hot side, whereas the $q_{h}$ stands for the heat flow received by the hot side, and $q_{hc}$ denotes the heat that flows out of the hot side and towards the Peltier cell. These three variables are measured in power units W. Taking Equation (6) as the baseline and adding Equation (5), after some mathematical manipulation, the temperature dynamics in the hot side can be obtained as follows:

(7) $$\frac{dT_{hs}}{dt}=\frac{q_{hs}}{C_{h}}-\frac{T_{hs}}{C_{h}R_{h}}$$

In Equation (7), $C_{h}$ represents thermal capacitance, measured in $J{/}^{\circ }C$; whereas $R_{h}$ stands for thermal resistance and has ${}^{\circ }C/W$ as units. Based on Figure 6, the heat transfer analysis is performed for the cold side, where $q_{hc}$ is the input, $q_{co}$ is the output, and $q_{cs}$ is the heat stored in the cold side. This relation is modeled in Equation (8), where the variables are measured in units of power W.

(8) $$q_{cs}=q_{hc}-q_{co}$$

Considering Equation (8) as the guideline and by adding Equation (5), the temperature dynamics in the cold side can be represented as in Equation (9).

(9) $$\frac{dT_{cs}}{dt}=\frac{k_{p}a_{p}\left(T_{hs}-T_{cs}\right)}{x_{p}C_{p}}-\frac{q_{cs}}{C_{c}}$$

In Equation (9), the sub-index *p* refers to the Peltier thermoelectric module, whereas the sub-index *c* is used to designate the cold side. An experiment explores the temperature in both sides when excited with step-like signals to understand the degree of coupling between the heat and cold sides.

**Figure 7:**
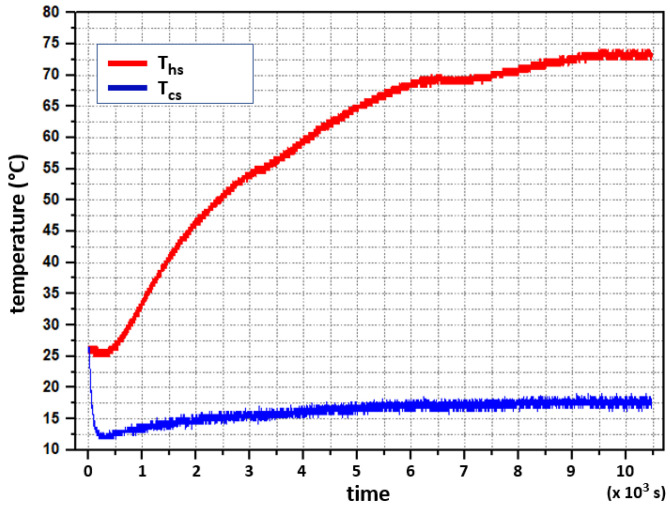
Experimental test. System’s response to a step input change in heating ($T_{hs}$) and cooling ($T_{cs}$) power from room temperature (26 ${}^{\circ }$C) up to 73 ${}^{\circ }$C in the hot side, and 17 ${}^{\circ }$C in the cold side.

The experiment consisted of the following: the system was initially at thermal equilibrium at $26{\;}^{\circ }C$. From this condition, two step signals were applied, one to the hot side and another one to the cold side. The heat resistors provided 52.01W, whereas the cooling Peltier modules received 49.72W of electrical power for cooling. The system’s response is shown in Figure 7. Both temperatures ($T_{hs}$ and $T_{cs}$) are modeled as first-order differential equations, as shown in Figure 7. Firstly, $T_{hs}$ increases exponentially and subsequently saturates and reaches $73{\;}^{\circ }C$ as supported by Equation (7); although no mathematical modeling for the transport delay in Figure 7 is included in Equation (7). Secondly, $T_{cs}$ loses heat faster through the cooling Peltiers, and this heat is quickly dissipated through the heatsinks. Subsequently, $T_{cs}$ rises up to $17{\;}^{\circ }C$ due to the effect of the released heat from the hot side and follows a first-order dynamic, as expected and modeled in Equation (9).

To register the temperature values in a personal computer (PC), an Arduino UNO development board was programmed for data acquisition, while data was displayed graphically using LabVIEW. A sampling period for data acquisition was set to T=1 s. The hot side reached $73{\;}^{\circ }C$ and the cold one $17{\;}^{\circ }C$ for steady-state values. After the abrupt temperature drop observed on the cold side at the beginning of the test, it is observed that for every $1{\;}^{\circ }C$ that the temperature increases on the cold side, the temperature of the hot side increases by about $10{\;}^{\circ }C$. Furthermore, the final value in both cases happened in more than one hour. Figure 7 evidences a delay-time phenomenon for the hot side at the beginning of a transient-time response. This behavior is caused by the heat capacity of the installed components, which determines the time constant of the heating (and cooling) process together with thermal resistances along the device.

The results illustrated in Figure 7 show a small interaction between the hot side and the cold side due to thermal conductivity. An improved heat insulation may reduce this effect. Since the cold plate was insulated from the heat irradiated from the hot side, it is believed that most of the heat is transferred through the thermoelectric module. Moreover, Equation (9) can be rewritten as Equation (10).

(10) $$\frac{dT_{cs}}{dt}=-\frac{q_{cs}}{C_{c}}-\frac{T_{cs}}{C_{c}R_{c}}$$

## 4. Closed-Loop System

Two discrete-time PI control algorithms are programmed in LabVIEW G code in a PC for temperature control. A controller regulates the temperature of the concrete slab or hot side of the TMUT, while the other controller regulates the temperature of the cold side of the TMUT; thus, a temperature difference can be kept constant over the TMUT. Controlling the temperature on the concrete slab emulates the heating effect of the sun rays while controlling the temperature on the cold side helps to emulate the cold side or shadow side of a concrete structure. Thus, by controlling the temperature on both sides, it will be possible to characterize the potential for energy conversion of any thermoelectric module attached to a concrete structure exposed to sunlight irradiation.

**Figure 8:**
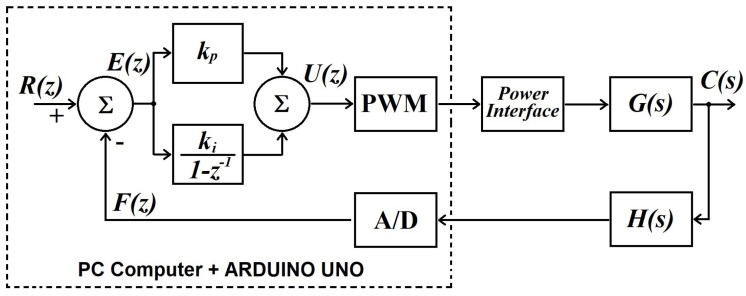
Block diagram representation of the controller’s design.

For this purpose, the design of each temperature controller focuses on working independently. The objective is to maintain specific desired temperature values on both sides of the TMUT. For the hot side, the system senses the concrete cube’s temperature, whereas on the cold side, the TMUT holds to an aluminum plate whose temperature is the variable to control. The controller’s design process includes a conceptual stage depicted by the block diagram regarding the main elements of the closed-loop system, as shown in Figure 8.

**Figure 9:**
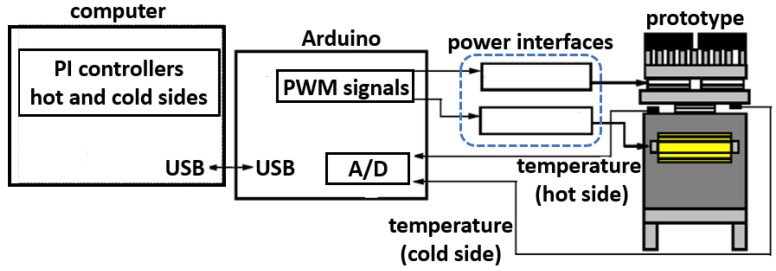
Block diagram of the implemented closed-loop control system.

Figure 8 shows the general block diagram that represents a single temperature control loop, which is valid for the temperature regulation of the hot and the cold side. There is a reference R(z), an error signal E(z), and the proportional-integral (PI) controller. For the purpose of this discussion, G(s) is known as the plant and represents the transfer function of either the hot side or the cold side, accordingly. The controller’s output is the signal U(z) that goes towards the continuous-time plant G(s). The process variable, C(s), is fed back through the block H(s), representing the sensor that measures the current temperature of either hot or cold sides, accordingly. The next phase is to implement the control system (Figure 9).

Figure 9 shows a block diagram of the implemented prototype for the temperature control of the hot side and cold side. The temperature sensor employed was an LM35 integrated circuit, one sensor per loop. The critical elements in Figure 9 are the PI controllers, which keep a constant temperature difference between the hot and cold sides. Based on the open-loop responses in Figure 7, the gains of the controller were tuned using the step-response tuning method by Ziegler-Nichols [24]. Furthermore, the computer hosted the control software, while an ARDUINO UNO^®^ board was used for data acquisition and to convey control signals to the power interfaces. Data captured by the ARDUINO board was transferred to the computer using a USB port configured as a serial port. The software LabVIEW created by National Instruments was employed to program a discrete-time linear control law. The following discrete-time PI control structure is valid for both temperature controllers:

(11) $$\frac{U_{x}\left(z\right)}{E_{x}\left(z\right)}=\frac{K_{px}\left(1-z^{-1}\right)+K_{ix}}{1-z^{-1}}$$

where $K_{px}$ is the proportional gain, and $K_{ix}$ stands for the integral gain. The subscript *x* could adopt *h* for hot or *c* for cold, depending on the controller temperature objective. Furthermore, the parameters of the differential equations were tuned and resulted in the following control equations:

Equation (12) is applied to the control of the hot side:

(12) $$U_{h}\left(k\right)=U_{h}\left(k-1\right)+\left(2.79\right)E_{h}\left(k\right)-2.773E_{h}\left(k-1\right)\;$$

Equation (13) is applied to the control of the cold side:

(13) $$U_{c}\left(k\right)=U_{c}\left(k-1\right)-\left(1.54\right)E_{c}\left(k\right)+1.18E_{c}\left(k-1\right)\;$$

The outputs of the controllers are sent to the ARDUINO UNO^®^ to adjust the duty cycle of two PWM signal generators. These signals become the inputs of the power interfaces which drive the actuators, namely, the heating resistors and the thermoelectric modules for the cooling of the cold side. Hot and cold temperatures are fed back through the sensors to close the loop. Keep in mind that two 10-bit analog-to-digital converters inside the ARDUINO UNO circuitry sample digitize the temperature signals for further processing. The sensing, processing, control signal generation, and driving actuator tasks repeat every sampling period (T=10 s).

Another component in the experimental tests is the human–machine interface (HMI) developed in LabVIEW 2009. The interface displays the current temperatures of the hot and cold side, the setpoints of the hot and cold temperatures, and the control signals $U_{h}$ and $U_{c}$. Moreover, the HMI allows to change diverse settings such as gains setting for the temperature controllers, sampling time, communication port, file path, and file name for data storage.

## 5. Results and Discussion

This section summarizes experimental results. First, simulation results are described, pertaining to open-loop and closed-loop temperature control. Second, plots are used in order to illustrate both controller performance and the electrical behavior of the TMUT, under steady temperature conditions.

### 5.1. Simulation—Closed-Loop Tests

**Figure 10:**
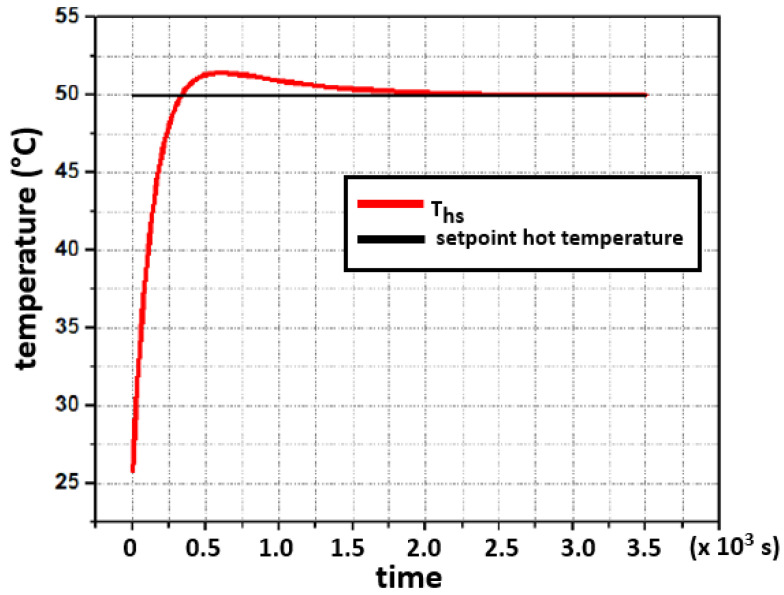
Simulated closed-loop response for a setpoint of $50{\;}^{\circ }C$ at the hot side with a starting temperature of $26{\;}^{\circ }C$.

**Figure 11:**
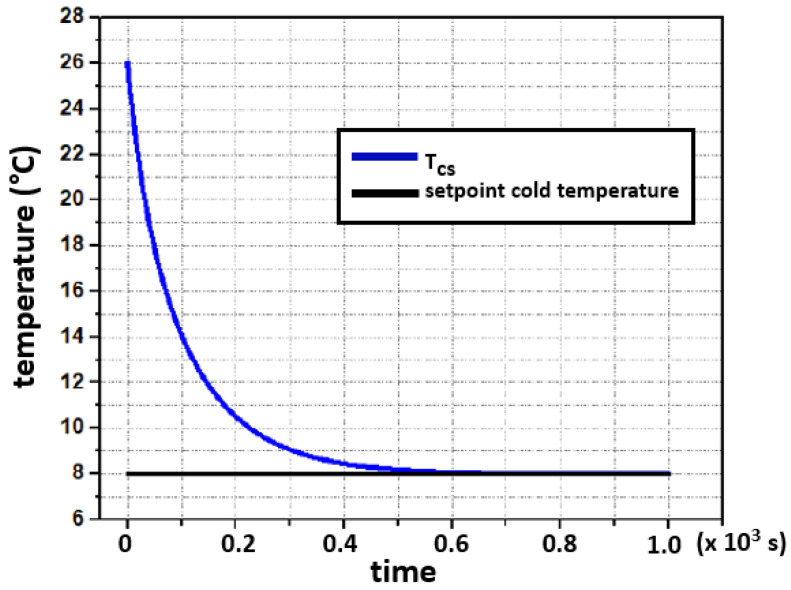
Simulated closed-loop response for a setpoint of $8{\;}^{\circ }C$ at the hot side with a starting temperature of $26{\;}^{\circ }C$.

The closed-loop simulation tests were developed in MATLAB-Simulink graphical programming tool and followed the concept shown in Figure 8. After the controller’s output, a saturation block limits the control signal within the power interface operating range. For the hot side, the range is [0, 65] W, and [0, 60] W for the cold side. In both cases, the initial system’s temperature was set to $26{\;}^{\circ }C$ and the setpoints were introduced as step signals, $50{\;}^{\circ }C$ and $8{\;}^{\circ }C$ for the hot side and cold side, respectively. The temperature dynamics, in both sides, are shown in Figure 10 and Figure 11.

In Figure 10 and Figure 11, it is observed that the setpoints are reached without any residual deviance after the settling process. The overshoot shown in Figure 10, is the result of a large delay, which is caused by the thermal coupling of the heater in combination with the temperature sensing of the concrete block (hot side). This delay is caused by the thermal resistances and heat capacities of involved components within that heat transmission path.

### 5.2. Experimental—Closed-Loop Tests

**Figure 12:**
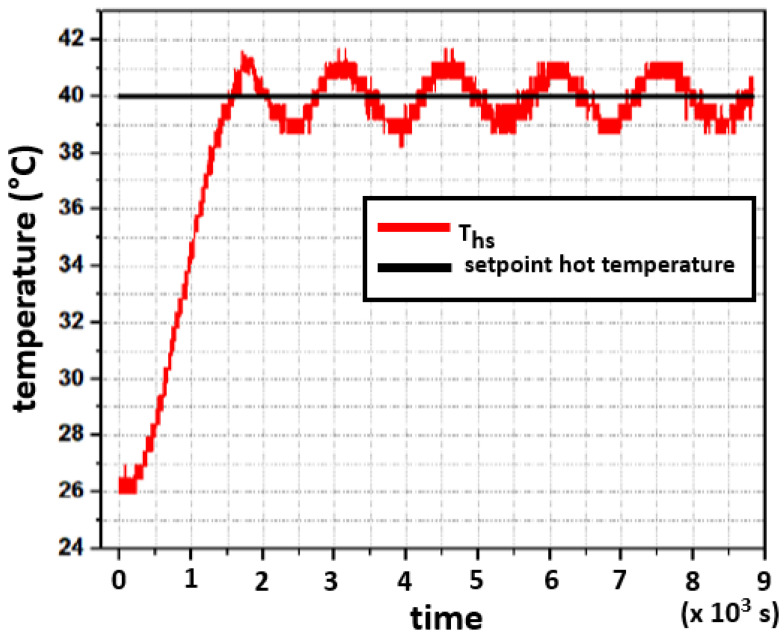
Experimental closed-loop response for the hot side. The setpoint is $40{\;}^{\circ }C$.

With the simulation tests performed, it is time to implement the control system. Figure 12 shows the response of the hot side when the setpoint is $40{\;}^{\circ }C$, and shows the oscillations around the setpoint, where the error peaked at $\pm 1.5{\;}^{\circ }C$, reducing to $\pm 1{\;}^{\circ }C$ after three cycles.

The initial temperature of the concrete cube is $26{\;}^{\circ }C$. The cube reached the desired temperature in 25 min (1500 s). An oscillation of $\pm 1{\;}^{\circ }C$ can be seen, and this phenomenon is maintained during the five cycles of the test, which ends in 2 h and 26 min, i.e., 8750 s. The lagging effect (not included in the modeling) causes the oscillation in the actuators (resistors HS1002R7).

**Figure 13:**
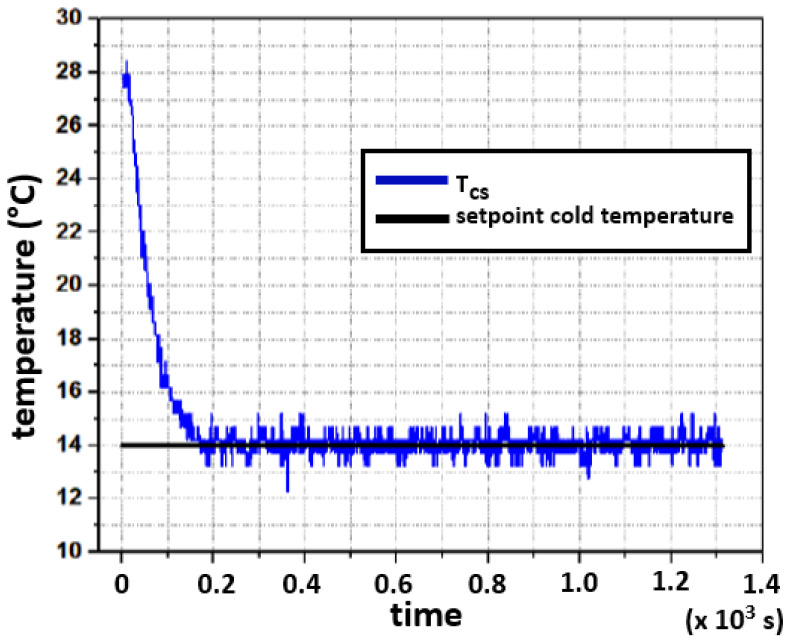
Experimental closed-loop response for the cold side. The setpoint is $14{\;}^{\circ }C$.

Figure 13 presents the response of the cold side, where the setpoint is $14{\;}^{\circ }C$. The initial temperature in the Peltier aluminum plate measured $28{\;}^{\circ }C$. The plate reaches the desired temperature in 3 min and 20 s. The closed-loop systems have a steady-state error of almost zero degrees, although the process variable keeps oscillating around the setpoint with an approximated error equal to $\pm 1{\;}^{\circ }C$. This is due to the long heat transport delay, previously described, which results in dead time for the controller. In this case, the result is an oscillating output of the closed loop control for the temperature of the hot side.

### 5.3. Stability

Stability was tested in the BIBO sense, i.e., bounded-input, bounded-output. The characterization system was subjected to different conditions to ensure that it remained stable within the temperature ranges in which it was designed to operate. As can be seen in Figure 7, when applying step-like signals of 52.01W to the hot side and 49.72W to the cold side, the power values are closed to the limits that the circuits can withstand, and the open-loop system reaches its steady state and remains there, which contributes to having BIBO stability in open-loop.

To apply BIBO criterion for stability in closed-loop, it is necessary to test the characterization system for different reference values on both faces and observe its behavior with the controllers.

**Figure 14:**
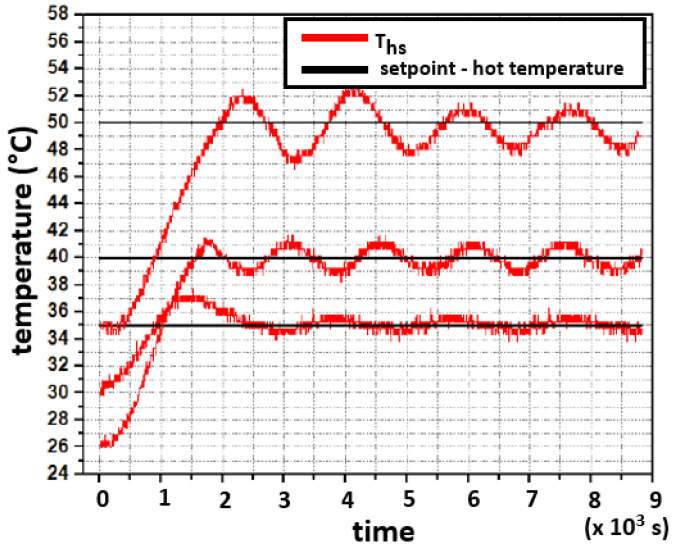
Three testing setpoints for the experimental closed-loop response for the hot side.

Figure 14 shows the response of the closed-loop system for the hot face when considering three different setpoints, each one of them with a unique starting temperature. The large thermal lag in the concrete slab leads to oscillations in the controller action [25,26,27]. It is proposed that a thinner concrete slab should be used to avoid these oscillations; however, in all three tests, the system remained stable.

**Figure 15:**
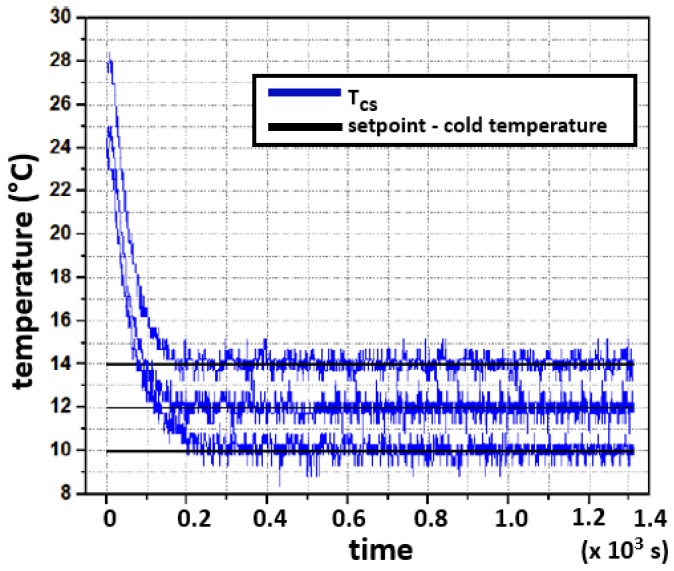
Three testing setpoints for the experimental closed-loop response for the cold side.

For the cold face, the same procedure is followed. Different reference values were set. It is observed in Figure 15 that the closed-loop system is stable for the three test scenarios.

### 5.4. Accuracy of Temperature Measurement

The application described in this work requires accurate temperature readings. The following actions help to reduce inaccuracies in the temperature readings. According to the manufacturer, the LM35 temperature sensor used in this work has an accuracy of $\pm 3/4{\;}^{\circ }C$ over the full operational range, from $-50{\;}^{\circ }C$ to $150{\;}^{\circ }C$. First, to achieve accurate and meaningful temperature readings, the LM35 sensor must be as close as possible to the interface between the TMUT and the concrete slab (hot face). The same indication must be followed for the cold face sensor. In both cases, the sensors are located at 1 cm from the TMUT. The position of both sensors is indicated in the diagram of Figure 3 and Figure 6. Second, applying a thin layer of a heat transfer compound between the sensor and the measurement surface ensures a proper thermal connection. Theoretically, the sensor measurements will produce voltage signals within the range of 200mV to 800mV. Electrical connections of the LM35 sensors should be kept as short as possible to reduce the effects of noise and voltage drop. Moreover, it is recommended that a twisted wire connection be used to reduce noise pickup.

### 5.5. Voltage Generation without Load (Open Circuit)

**Figure 16:**
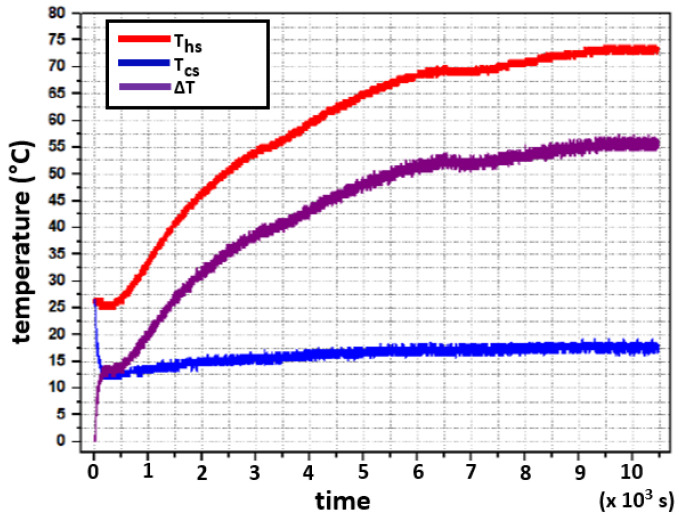
Temperature difference between the cold and hot sides.

The voltage generation depends on the temperature difference, or Δ*T*, between the hot and cold sides. Figure 16 shows the temperatures that were reached on both sides and the temperature difference between them. Because the experiment started in thermal equilibrium, the Δ*T* starts at zero and increases over time, according to the dynamics that each face follows. The maximum value of Δ*T* that was obtained is $55.86{\;}^{\circ }C$ at steady state, and it was reached in a time frame of 2 h and 38 min (9500s).

**Figure 17:**
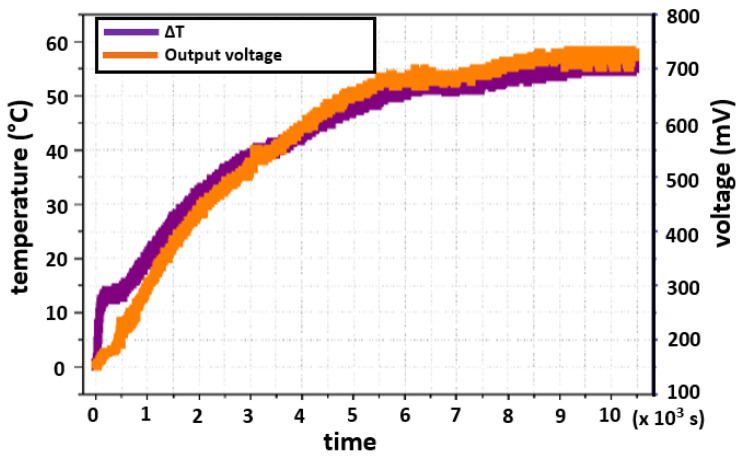
Output voltage and temperature difference.

Figure 17 depicts the temperature difference and the generated voltage of the thermoelectric module. As mentioned above, the maximum measured Δ*T* was 55 °C, for which the module generates 730mV.

### 5.6. Voltage Generation with Load (Closed-Circuit)

**Figure 18:**
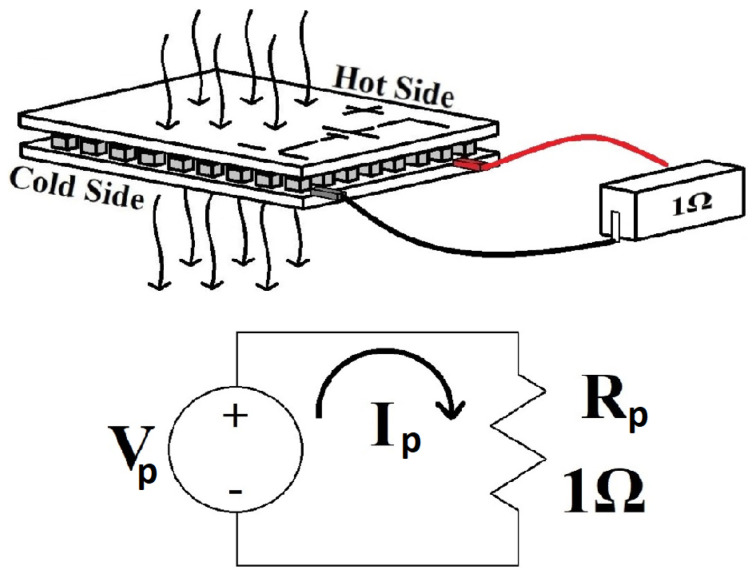
The electric diagram represents the measured voltage and current provided by the thermoelectric module.

The thermoelectric module generates the voltage and currents according to the schematic in Figure 18. The diagram shows the voltage source Vp (the TMUT output), Rp, a reference resistor (1Ω, 1% tolerance, precision resistor, measured at 0.99927Ω), and the electric current Ip. The TMUT chosen for this experiment has an internal resistance of 1Ω, according to the datasheet of the device. Thus, tests were conducted closely to the condition of maximum power transfer. A digital multimeter with a 3-1/2 digits reading screen and 10 MΩ input impedance, manufactured by STEREN, model MUL-605, was used to measure the voltage of the TMUT. An impedance bridge, manufactured by GW INSTEK, model LCR-800, was used to characterize the Rp values.

**Figure 19:**
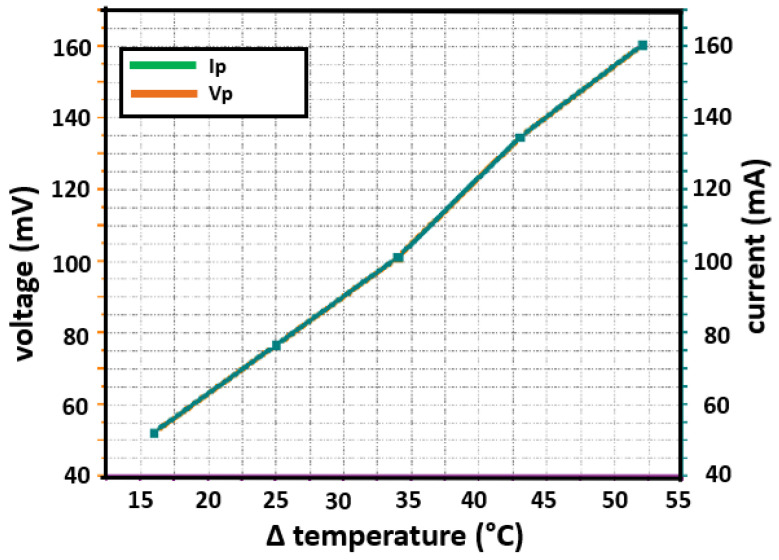
Output voltage Vp and current Ip for an Rp = 1Ω.

Figure 19 depicts the response of a TMUT to a set of five temperature settings at steady state. These temperature settings, correspond to five Δ*T*, range from $16{\;}^{\circ }C$ up to $52{\;}^{\circ }C$. As seen in Figure 19, the higher the Δ*T*, the more voltage and current are generated. Since Rp = 1Ω, the voltage and current values overlap. Table 3 lists the measurements of voltage and current values taken at specific Δ*T* values.

### 5.7. Electrical Power

**Figure 20:**
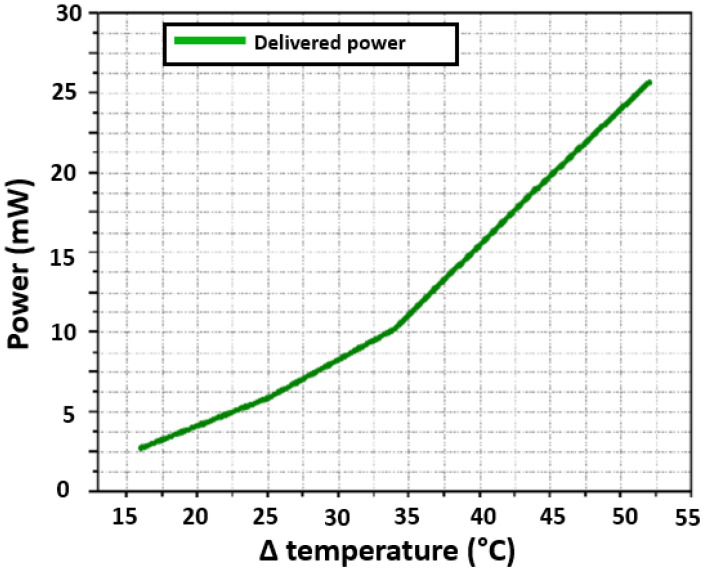
Delivered power by the thermoelectric module.

Figure 20 shows the relationship that exists between the temperature difference and the electrical power delivered by the thermoelectric module. In addition, it shows that at $34.5{\;}^{\circ }C$ there is an inflection point, and from there, the electrical power increases faster. At a temperature difference of $52{\;}^{\circ }C$, the electrical power delivered reaches 25.7148mW. Such value is the maximum that the characterization system can achieve at present. The small inflection point in Figure 20 may be due to a nonlinear response of the thermoelectric module. This nonlinear response can also be seen in Figure 19. Figure 20 shows the relationship that exists between the temperature difference and the electrical power delivered by the thermoelectric module. Since power is a quadratic function of current or voltage, the nature of the inflection point is concealed by this.

### 5.8. Discussion

Most heat to electric energy conversion schemes focus on high-temperature and high-pressure steam production. It is also the case for some research facilities dedicated to investigating hybrid optical–mechanical methods to concentrate solar radiation to transform water into steam for energy generation based on steam turbines [28]. Such methods are prone to high maintenance costs, require continuous supervision, and have a limited operational time, usually in daylight hours. In this work, the authors propose that the heat naturally stored in concrete in many structures can be transformed into electrical energy through thermoelectric energy conversion. Those structures lay down in the landscape, storing heat energy directly from solar irradiation in highways, bridges, dams, buildings, and other large structures; refitting those structures with thermoelectric modules may not affect the ecosystems any further, such as in the case of solar panels and wind turbines, which take up acres of land that must be cleared out of the vegetation to install.

In order to produce energy efficiently from thermoelectric modules, it is essential to investigate what energy conversion efficiency to expect from different combinations of concrete and thermoelectric modules. Further research work is needed to improve mechanical bonding, efficient energy transfer, and conversion to prolong energy production and improve investment returns. Thus, this work proposes a starting point by providing a first attempt to characterize the thermoelectric modules in installation conditions with heat exchanging components made from concrete.

For example, for conditions in the city of Hermosillo, Mexico, located at 29${}^{\circ }$05${}^{\prime }$56${}^{\prime \prime }$ N and 110${}^{\circ }$57${}^{\prime }$15${}^{\prime \prime }$ W, it can be assumed that a concrete roof of a house in summer can reach more than $60{\;}^{\circ }C$. Therefore, the difference in temperatures of $43{\;}^{\circ }C$ is taken, corresponding to a temperature of $60{\;}^{\circ }C$ on the hot face and $17{\;}^{\circ }C$ on the cold one. Under these conditions, a single thermoelectric module produces 134.7 mV with a current of 134.75 mA for a load of 1Ω, thus supplying an electrical power of 18.15 mW.

It is necessary to point out that the present location of the heating resistors may cause undesired heat flows that may affect the performance of the TMUT. It has also been observed that the thermal isolation and the device geometry may be improved to avoid heat loses by convection.

## 6. Conclusions and Further Work

This work describes a prototype to characterize thermoelectric modules attached to a concrete slab. The prototype characterizes different operational conditions for thermoelectric modules to convert heat energy into electrical energy when attached to concrete structures. The proposed prototype demonstrated a thermoelectric module to produce power under a controlled temperature difference. The results illustrate that under the conditions described in the experiment, the approximate time constant of the rig is 2500 s, or 40 min. Other variables and parameters remain to be characterized and considered in the experiments. Among these are heat losses from concrete, heat losses on the aluminum plate, various thermal resistances in the TMUT and surrounding the TMUT, etc. The prototype stores and releases energy, closely resembling the response of a first-order system, as can be seen in Figure 15.

Concrete stores large amounts of energy due to its large specific heat, ranging from 850 J/kg·K up to 1050 J/kg·K, depending on the mixtures of other aggregates [29,30,31,32]. The thermoelectric module shows low efficiency. For example, for a temperature difference of $50{\;}^{\circ }C$, the average energy stored in a cube of $1000\;{\mathrm{cm}}^{3}$ of concrete is 5220J. Although thermoelectric modules designed for energy generation have improved efficiency over those thermoelectric modules manufactured for cooling, efficiency is lower than solar cells. However, energy-efficient sensors require only small amounts of energy, which a thermoelectric module can supply.

The main advantage of thermoelectric modules for energy generation is that energy is stored in concrete, while solar cells must store energy in batteries. Batteries represent a significant part of the investment in photovoltaic energy infrastructure with a moderated replacement frequency. On the other hand, the heat stored in concrete will remain for hours after the sunlight has resided, meaning that power will still be available to power up sensors and other devices close to or within the concrete structure. Therefore, it can be reasonable to assume that a combination of solar panels and thermoelectric modules will prolong battery life and improve energy efficiency. Many concrete structures can be found in the countryside landscape and the inner cities worldwide. The energy stored in those concrete structures can power different sensor devices, communication devices, data transfer devices, smart highway sensors, and provide energy for many other applications.

Further work is required to compensate the oscillations on the temperature of the hot side. PI controllers are affected by long heat lags, which are present in the concrete slab. A downsizing of the concrete slab thickness, along with the implementation of a Smith predictor algorithm, are proposed in order to reduce the oscillations. The location of the heating resistors must be reconsidered in order to minimize the effects of stray heat flow in the TMUT.

An attractive research line for future exploration is to study how the load of a thermal–electric device affects its maximum power. Potential maximum power point tracking strategies to consider are those in [33,34,35].

## Figures and Tables

**Table 1:** List of mechanical components. Description, dimensions, and quantity of the materials that were used for the construction of the prototype. Elements 1 to 11 are the same ones as those in Figure 2.

#	Description	Dimensions (cm)	Qty
1	Cooling fans 12 V, 0.4 A	6.0×6.0×2.5	2
2	Heatsink	12.6×6.3×4.1	1
3	Peltier cell TEC1-12715	12.6×6.3×4.1	2
4	Aluminum plate (cold element)	12.3×11.2×0.5	1
5	Thermoelectric Module Under Test (TMUT) TEC1-12710	4.0×4.0×0.4	1
6	Concrete cube (hot element)	7.93×7.93×7.93	1
7	Heating resistor HS100 2R7	6.52×2.73×2.41	2
8	Aluminum base	13.0×13.0×1.27	1
9	Threaded rod 10–24	0.4826 D, 16 L	4
10	Insulating plate	3.2 D, 0.12 L	4
11	Supporting rod	1.9 D, 5.0 L	4
12	Polyurethane foam	as needed	1 can
13	Threaded rod nuts 10–24	0.7 D, 0.32 L	4
14	Bolts 4–40 for the insulating plates	0.2844 D, 0.635 L	16
15	Bolts 4–40 for the heatsink	0.28 D, 1.27 L	4
16	Bolts 4–40 for the cooling fans	0.2844 D, 5.08 L	8

**Table 2:** List of electronic components. Designations and quantity of the electronic components used in each thermoelectric module driver circuit used for cooling.

#	Description	Designation	Qty
1	Optocoupler	4N25	1
2	Op-amp	LM741	1
3	Power op-amp	OPA541AP	2
4	Resistors R1	0.15Ω	1
5	Resistors R2	0.15Ω	1
6	Decoupling capacitors	1 μF	6
7	Compensation capacitors	20 pF	2
8	Gain adjusting potentiometer	1 kΩ	1
9	Copper clad board	RF4, 1/2 oz	1

**Table 3:** List of measurements of electrical power generation under specific Δ*T* values.

Hot Face	Cold Face	Δ *T*	Voltage	Current
	${}^{\circ }$ **C**		**mV**	**mA**
30	14	16	52.19	52.228
40	15	25	76.6	76.656
50	16	34	101.1	101.174
60	17	43	134.7	134.748
70	18	52	160.3	160.417

## Data Availability

Not applicable.
